# Renal anaemia treatment in haemodialysis patients in the Central and Eastern European countries in everyday clinical practice follow-up

**DOI:** 10.1007/s11255-012-0303-0

**Published:** 2012-11-08

**Authors:** Jolanta Malyszko, Maciej Drozdz, Agnieszka Zolkiewicz, Boleslaw Rutkowski

**Affiliations:** 1 Department of Nephrology and Transplantology, Medical University, Zurawia 14, 15-540 Bialystok, Poland; 2 Department of Nephrology, Collegium Medicum, Jagiellonian University, Kraków, Poland; 3 Roche Pharmaceutical Company, Warsaw, Poland; 4 Department of Nephrology, Transplantology and Internal Diseases, Medical University, Gdańsk, Poland

**Keywords:** Anaemia, Central and Eastern Europe, Haemodialysis, Cardiovascular events

## Abstract

**Background:**

Chronic kidney disease is almost always accompanied by anaemia. Erythropoietin-stimulating agents (ESA) can increase haemoglobin concentration and thus reduce the frequency of anaemia-related complications including the cardiovascular events.

**Aim:**

The aim of the study was to collect prospective data on 12-month standard ESA therapy used in haemodialyzed patients in selected CEE countries as well as on cardiovascular complications, iron status and anaemia treatment.

**Patients and methods:**

Fifty centres in 3 countries participated in the study. A group of 398 haemodialysed stable patients (M-231, F-167) aged 19–90 years (57.5 ± 14.7) on standard ESA therapy for chronic renal anaemia were recruited. Twelve-month prospective data on iron parameters, ESA therapy and cardiovascular events were collected. The use of iron, folic acid and blood transfusions were also assessed. Patient were divided into three groups according to ESA therapy start: group A—patients who received ESA after start of haemodialysis, group B—patients who received ESA within 3 months from the day of first haemodialysis and group C—patients who had received ESA more than 3 months before haemodialysis. Chi^2^ test for qualitative data and Kruskall–Wallis test for quantitative data with *p* < 0.05 were used in statistical analysis.

**Results:**

At prestudy period, the mean weekly dose of ESA in group C was statistically lower than in the remaining two groups (3,823 ± 3,169 vs. 5,276 ± 2,915 and 6,427 ± 3,441 units/week, *p* < 0.001), but during prospective phase of the study the doses did not differ among groups A, B and C. No major fluctuation of ESA administration schedule was observed during the study in the groups; however, at majority of visits, the mean frequency of ESA administration in group C was statistically higher than in groups A and B. At baseline visit, the haemoglobin concentration in group A patients (10.86 ± 1.34 g/dL) was slightly lower than in group B (11.26 ± 1.43 g/dL) and group C (10.98 ± 1.35 g/dL) (*p* = 0.025), but at subsequent visits these differences disappeared and mean haemoglobin concentration was stable around 11 g/dL. Ferritin concentration increased from 280 ± 241 at baseline to 506 ± 405 at month 12, and no important differences in the groups were observed. The other haematological parameters (haematocrit, iron concentration) remained stable during the entire study. The frequency of blood transfusion and total volume of blood in group C were lower than in groups A and B. During the prospective 12-month follow-up, 23 (5.8 %) of the patients died and 35 (8.8 %) were transplanted. No differences in death or transplantation rate were observed among groups A, B and C. The number of patients with adverse events, serious adverse events or drug-related adverse events in all groups was similar. In conclusion, ESA therapy increased haemoglobin concentration and no major differences in haematological parameters among the groups were observed during the entire study irrespective of early versus late start. Mortality, cardiovascular events or other adverse events were similar among the groups during the observation period; however, the limitation of the study is the sample size.

## Introduction

Anaemia has remained one of the most characteristic and visible manifestations of chronic renal failure for over 150 years. Typically, it is a normocytic and normochromic anaemia with bone marrow of normal cellularity. Anaemia has been defined as a reduction in one or more of the major red blood cell measurements: haemoglobin concentration (Hb), haematocrit or erythrocyte count. The pathogenesis of anaemia of chronic kidney disease is multifactorial [1]. Although inadequate production of erythropoietin is the most important factor in the pathogenesis of anaemia in chronic kidney disease, other factors play a role and contribute to mild anaemia that is often present despite the use of recombinant human erythropoietin or other erythropoiesis stimulating agents (ESA). Renal anaemia has a number of potentially deleterious effects, including impairment of tissue oxygen delivery, increased cardiac output and left ventricular hypertrophy predisposing to congestive heart failure cognitive decline, sexual dysfunction and depression of immune responsiveness [2]. Moreover, cardiovascular disease is a leading cause of death in both chronic kidney disease (CKD) and dialysed patients [3, 4]. Silverberg et al. [5] proposed the term ‘cardio-renal anaemia syndrome’ to stress the importance of mutual relations in the populations suffering from CKD and/or CHF. Administration of ESA to increase Hb concentrations from lower values to >10–11 g/dL significantly reduces the cardiovascular complications of renal anaemia and reduces the frequency of chronic heart failure and hospitalization among predialysis and dialysis-dependent patients [6, 7]. Recent randomized trials (CHOIR, CREATE and TREAT) [8–10] clearly showed that targeting higher Hb levels in CKD has been linked with increased morbidity and mortality particularly from cardiovascular causes. There might have been several mechanisms of such unexpected harm, besides increased viscosity through an increase in haematocrit. Also a recently published study in Veteran Administration predialysis patients showed lowered risk of hospitalization (by 17 %) and of transfusion by 29 % when ESA were initiated early (Hb 10–11 g/dL) versus late (Hb level 9–9.9 g/dL) [11].

We designed a multicenter, open-label, observational study to assess the impact of early versus late referral on the effectiveness of renal anaemia treatment with ESA in 3 groups of haemodialysis patients (treated less than 3 months of ESA before HD, treated more than 3 months of ESA before HD and not treated with ESA before HD) with special interest on the medical history (early/late referral, cardiovascular events) and safety in a large Central and Eastern European countries (CEE). We presented the baseline data of this study previously [12]. In the 12-month follow-up, we looked at the current renal anaemia management, iron parameters and haemoglobin in haemodialysed patients in relation to the early or late referral in CEE countries in everyday clinical practice. Additionally, we assessed the number of cardiovascular (CV) events, deaths and hospitalization for CV or other events.

## Patients and methods

Patients suitable for the study should fulfil the following inclusion criteria: stable haemodialysed patients with renal anaemia who are no longer than 12 months on haemodialysis and who are on ESA therapy; patients who had no fluctuation of Hb more than 2 g/dL/month within the last 3 months of ESA treatment; aged ≥18 years; who gave signed the written informed consent. Fifty public centres in 3 countries participated in the study, predominantly non-academic. A total of 398 haemodialysed patients (M-231, F-167) aged 19–90 years (57.5 ± 14.7) on standard ESA therapy for chronic renal anaemia were recruited. The primary kidney diseases were the following: diabetic nephropathy (*n* = 75; 18.84 %), glomerulonephritis (*n* = 64; 16.08 %), hypertensive nephropathy (*n* = 61; 15.33 %), interstitial nephritis (*n* = 79; 19.85 %), ADPKD (*n* = 38; 9.55 %) and other (*n* = 33; 8.29 %) or unknown (*n* = 48; 12.06 %). Concomitant diseases were as follows: diabetes (*n* = 102; 25.63 %), hypertension (*n* = 354; 88.94 %), coronary heart disease (*n* = 113; 28.39 %), chronic heart failure (*n* = 115; 28.89 %), left ventricular hypertrophy (*n* = 192; 48.24 %), peripheral occlusive disease (*n* = 50; 12.56 %), dyslipidaemia (*n* = 171; 42.96 %), secondary hyperparathyroidism (*n* = 142; 35.68 %), while recorded prestudy cardiovascular event included myocardial infarction (*n* = 41; 10.30 %), coronary artery bypass grafting (*n* = 7; 1.76 %), percutaneous coronary interventions (*n* = 14; 3.52 %), stroke (*n* = 19;4.77 %) and transient ischemic attack (*n* = 19; 4.77 %).

The following prospective parameters had been collected during the study and were analysed: Hb concentration, haematocrit, iron concentration, ferritin, blood pressure, ESA therapy (dose and frequency), medications (including iron supplementation), all major CV events (myocardial infarction-MI, percutaneous coronary interventions-PCI, coronary artery bypass grafting, stroke, transient ischemic attacks-TIA), adverse events, deaths. The data were collected using electronic data capture system. Data were verified for logical and medical consistency according to the instructions provided in CTAP. In case of inconsistencies, the data query was generated and was sent for investigator’s review. A part of problems was resolved; however, some queries were not addressed by investigators. Unresolved issues are related to missing data allowing to allocate patient to specific study group (15 patients), discrepancies between laboratory results and acceptable range of results, and AE description. All available and logically acceptable data were analysed.

Patients were divided into three groups:

Group A: Patients not treated with ESA before the start of dialysis
Group B: Patients who had started ESA therapy within three months from the start of dialysis
Group C: Patients who had started the ESA therapy more than three months before the start of dialysis

Comparisons were performed for all patients allocated to one of the groups. Patients without allocation were excluded from analysis.

### Statistical analysis

The data recorded in the database were provided for analysis in EXCEL format. The data were analysed in SAS statistical software. χ^2^ test was used for analysis of qualitative data and Kruskall–Wallis test for quantitative data. *P* value ≤0.05 was defined as statistically significant.

## Results

### Baseline patient’s characteristics

The study was performed on 398 patients from 50 centres in Latvia, Poland and Serbia. There were 231 (58 %) males and 167 (42 %) females aged 19–90 years (57.5 ± 14.7). A total of 269 patients completed 12 months of therapy. The reasons for study discontinuation were provided previously [12]. Based on the start dates of ESA and dialysis therapy, patients were allocated to groups A, B and C:

- Group A—180 subjects (106 males, 74 females) aged 19–86 years (56.2 ± 14.4 years).
- Group B—164 subjects (94 males, 70 females) aged 19–90 years (57.6 ± 14.9 years).
- Group C—39 subjects (21 males, 18 females) aged 20–86 years (61.8 ± 16 years).

No statistically significant differences among groups A, B and C according to age and sex distributions were found. Fifteen patients could not be classified into any of the groups and were excluded from comparative analysis. Groups A, B and C did not differ according to baseline: arterial blood pressure, body weight or height and BMI.

### Prospective laboratory results during study visits

Haematology assessment was done at each study visit. It consisted of evaluation of the following parameters: haemoglobin, haematocrit, serum iron and ferritin concentration.

#### Haemoglobin concentration

**Fig. 1:**
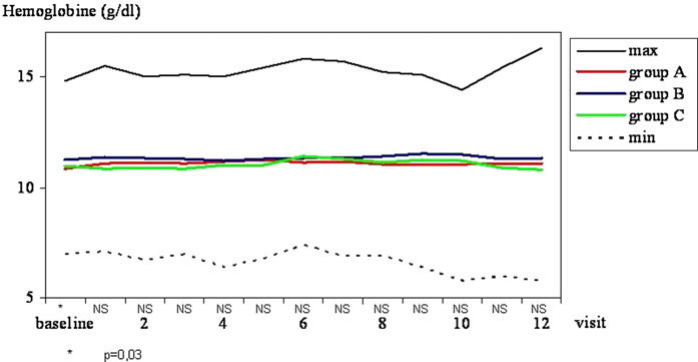
Haemoglobin concentration (g/dL). Maximal and minimal values in all patients tested and mean values in *groups A, B* and *C* at subsequent visits

Except from baseline evaluation, the mean haemoglobin concentration did not differ significantly among the groups A, B and C (Fig. 1).

#### Haematocrit

**Fig. 2:**
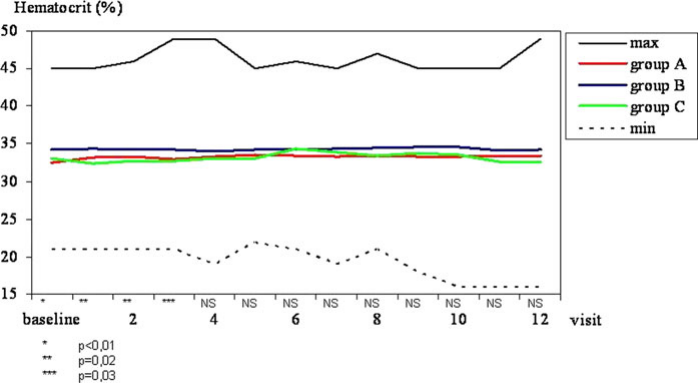
Maximal, minimal and mean haematocrit in all patients tested in *groups A, B* and *C* at subsequent visits

Mean haematocrit in group B patients from baseline to visit 3 was statistically higher than in the remaining two groups. Mean haematocrit in all three groups was not statistically different starting from visit 4 onwards (Fig. 2).

#### Serum iron concentration

**Fig. 3:**
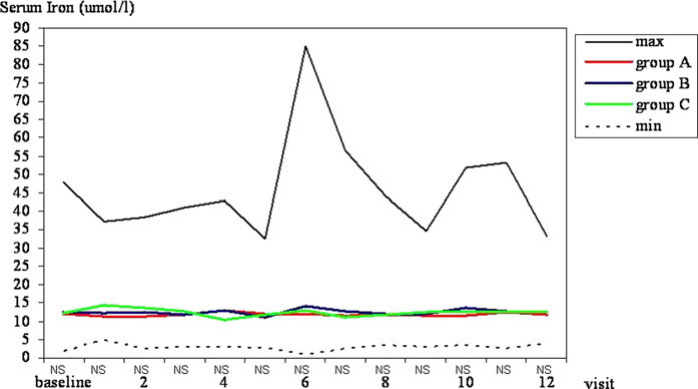
Serum iron concentration (μmol/l). Maximal and minimal values in all patients tested and mean values in *groups A, B* and *C* at subsequent visits

Mean serum iron concentration in all three groups was stable during all visits. No statistically significant differences in serum iron were observed at any time of the study (Fig. 3).

#### Ferritin concentration

**Fig. 4:**
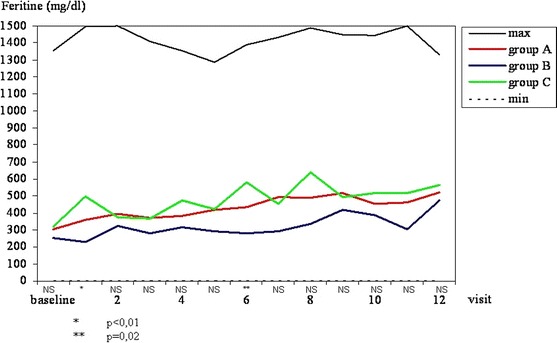
Ferritin (mg/dL). Maximal and minimal values in all patients tested and mean values in *groups A, B* and *C* at subsequent visits

In all three groups, ferritin concentration tended to increase during the study. Except for visits 2 and 6, there were no statistically important differences in ferritin concentration among groups A, B and C (Fig. 4).

### Prospective concomitant treatment for anaemia

**Fig. 5:**
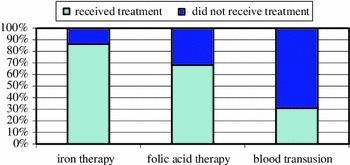
Concomitant treatment for anaemia in all study patients. There were no statistically significant differences in the rate of patients on iron or folic acid therapy nor in those who received blood transfusion among *groups* *A, B* and *C* (Fig. 6)

**Fig. 6:**
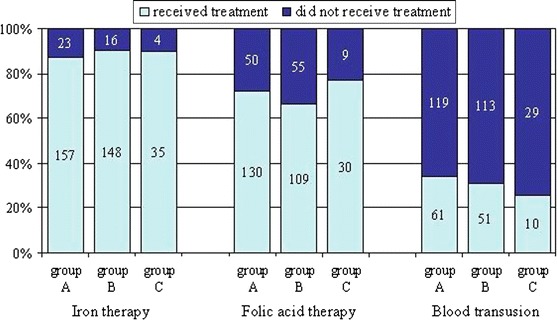
The rate of patients on iron therapy, folic acid therapy or patients who received at least one blood transfusion during the study in *groups A, B* and *C*

Concomitant treatment for anaemia consisted of iron, folic acid and blood transfusions. Majority of patients received iron therapy (86, 7.5 % of them were given oral iron, the remaining intravenous iron) and folic acid therapy (68 %). Blood transfusions were performed in 31 % of subjects (Fig. 5, 6).

The mean number of blood transfusion per patient in group A was statistically significantly higher than that of the remaining two groups (2.7 ± 2.45 vs. 1.75 ± 1.28 and 1.4 ± 0.7). The mean of the total volume of blood transfused to one patient in group A was significantly higher than that of groups B and C (respectively, 1,329 ± 1,201 vs. 940 ± 803 and 590 ± 248 mL, *p* = 0.01). There was no statistically significant difference in the number of patients treated with iron, folic acid or given blood transfusion between groups (*p* = 0.95, *p* = 026, and *p* = 0.56, respectively).

When we look at the haematological status of the patients, we found that 17 % of the patients had Hb <10 g/dL during the study period (ranging from 14 to 24 %), whereas 7 % had Hb >13 g/dL (ranging from 0 to 11 %). To correct the calculated EPO resistance for differences in Hb levels, EPO resistance index (ERI) was determined as the ratio EPO/Hb [defined as weekly EPO dose (U/kg b.w.) divided by Hb level (g/dL)]. We found that ERI was 9.35 ± 7.37.

We also looked at the CRP (assessed by laboratories using low-sensitivity method) and found that median CRP was 4.7 (0–622 mg/L) and there were no statistically significant changes between visits. Of the patients, 26 % had CPR >10 mg/L, whereas 40 % of the patients had CRP >6 mg/L .

### Prospective concomitant treatment for hypertension and CHD

Statins were administered to 34 % of patients, and ACE inhibitors or AT1 blockers were administered to 34 % of patents as well. Most patients (82 %) received other antihypertensive medication (Table 1). The rate of patients on ACE or AT1 blockers in group B was statistically higher than that in groups A and C.

**Table 1:** Summary of hypertension and CHF treatment

	Total (*N* = 398)		Group A (*N* = 180)		Group B (*N* = 164)		Group C (*N* = 39)		*p*
	*N*	%	*N*	%	*N*	%	*N*	%	
No. of patients receiving statins	137	34.4	60	33.3	60	36.5	17	43.6	NS
No. of patients receiving ACE inhibitors/AT1 blockers	135	33.9	52	28.9	69	42.1	14	35.9	0.038
No. of patients receiving other antihypertensive medication	327	82.1	147	81.7	143	87.2	36	92.3	NS

### Prospective other concomitant medication

Totally 827 concomitant medications were reported in the database. The most frequently administered concomitant medication was vitamin B12 (24.6 %), acetylsalicylic acid (18.6 %) and Calcium carbonicum (18.1 %).

### Prospective blood pressure

**Fig. 7:**
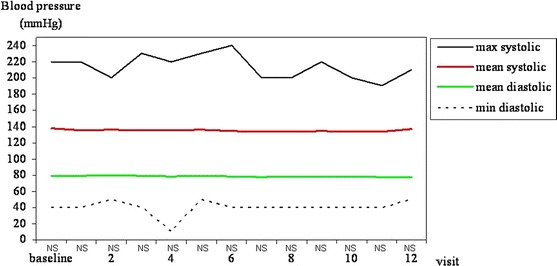
Predialysis blood pressure (min diastolic, mean diastolic, mean systolic and max systolic) during the subsequent study visits in all patients participating in the study

The mean systolic and diastolic blood pressure in the whole group of patients did not change during the study period (Fig. 7). Neither the systolic blood pressure nor the diastolic blood pressure differs among groups A, B and C at any visit.

The mean dialysis time was 705 ± 94 min. Group A patients were on dialysis statistically significantly longer than patients from groups B and C (respectively, 721 ± 92 vs. 691 ± 92 and 704 ± 102 months, *p* = 0.02). No changes in dialysis adequacy (KT/V) were observed during the study period. The groups A, B and C did not differ according to dialysis adequacy at any study visit.

### Prospective adverse events excluding common dialysis-related AE

The protocol predefined the adverse events of special interest. Within 12 months of the study, 8 episodes of myocardial infarction in 7 patients were recorded. One patient had coronary artery bypass, and 3 patients had 3 episodes of percutaneous coronary intervention. Stroke occurred in 6 patients including one patient with two episodes. The frequency of these adverse events did not differ among groups A, B and C (Table 2). Other AE were reported in 174 (43.7 %) patients. A total of 486 AE were reported. Mean number of adverse events per patient was 1.22. The most frequent adverse events were pneumonia (16 episodes), urinary tract infection (16 episodes), fever (12 episodes) and bronchitis (10 episodes). No differences in the number of patients with AE and the mean number of AE episodes among groups A, B and C were noted (Table 2). A total of 187 serious adverse events were reported in 99 patients (Table 3). Mean number of the serious adverse events episodes in all patients was 0.47. There were no differences in the number of patients with the episode or mean number of episodes among groups A, B and C (Table 4). Investigator assessed that in 33 patients the event was study drug related. Neither differences in the number of patients with this kind of episode nor the mean number of episodes were observed among groups A, B and C (Table 2). Twenty-three patients died and 35 patients received kidney transplantation. The death rate and transplantation rate in all groups did not differ (Table 3).

**Table 2:** Number of adverse events

	Total (*N* = 398)			Group									p*
				A (*N* = 180)			B (*N* = 164)			C (*N* = 39)			
	Patients with episode	Number of episodes	Mean number of episodes	Patients with episode	Number of episodes	Mean number of episodes	Patients with episode	Number of episodes	Mean number of episodes	Patients with episode	Number of episodes	Mean number of episodes	
Myocardial infarction (MI)	7	8	0.02	4	5	0.028	3	3	0.018	0	0	0	NS
Coronary artery bypass graft—without MI	1	1	0.002	1	1	0.006	0	0	0.0	0	0.0	0	NS
Percutaneous coronary intervention—without MI	3	3	0.007	1	1	0.006	2	2	0.012	0	0	0	NS
Stroke	6	7	0.017	3	3	0.017	1	1	0.006	2	3	0.08	NS
Transient ischemic shock	0	0	0	0	0	0	0	0	0	0	0	0	
Other (all AE except dialysis-related events)	174	486	1.22	91	256	1.42	65	179	1.09	18	51	1.31	NS

* χ^2^ for number of patients with the episode

**Table 3:** Number of serious and drug-related adverse events

	Total (*N* = 398)			Group									p*
				A (*N* = 180)			B (*N* = 164)			C (*N* = 39)			
	Patients with episode	Number of episodes	Mean number of episodes	Patients with episode	Number of episodes	Mean number of episodes	Patients with episode	Number of episodes	Mean number of episodes	Patients with episode	Number of episodes	Mean number of episodes	
Serious adverse events	99	187	0.47	54	107	0.59	34	56	0.34	11	24	0.62	NS
Drug-related adverse events	28	33	0.08	16	16	0.089	11	14	0.09	1	3	0.08	NS
Death	23	23	0.06	13	13	0.07	8	8	0.05	2	2	0.05	NS
Transplant	35	35	0.09	20	20	0.11	11	11	0.07	4	4	0.10	NS

* χ^2^ for number of patients with the episode

**Table 4:** The frequency of the AE recorded as other (the AE with the overall occurrence rate >2.5 % are included)

Major adverse events	Total (*N* = 398)			Group									p*
				A (*N* = 180)			B (*N* = 164)			C (*N* = 39)			
	Patients with episode	Number of episodes	Mean number of episodes	Patients with episode	Number of episodes	Mean number of episodes	Patients with episode	Number of episodes	Mean number of episodes	Patients with episode	Number of episodes	Mean number of episodes	
Pneumonia	15	16	0.04	10	11	0.06	3	3	0.02	2	2	0.05	NS
Urinary tract infection	11	16	0.04	5	6	0.03	6	10	0.06	0	0	0.00	NS
Fever	8	12	0.03	5	9	0.05	3	3	0.02	0	0	0.00	NS
Acute bronchitis	7	10	0.03	2	4	0.02	5	6	0.04	0	0	0.00	NS

* χ^2^ for number of patients with the episode

## Discussion

For the first time, we demonstrated that in EEC majority of patients with chronic kidney disease receive ESA therapy shortly before or after the start of dialysis treatment. In addition, we have found that despite iron supplementation (86 % of the studied population), serum iron remained stable as well as the total weekly dose of NeoRecormon (median dose for group A was 6,000 IU per week at baseline and 4,000 IU after 12 months, for group B 5,000 IU and 4,250 IU, for group C 6,000 IU and 6,000 IU per week, respectively). Except for visits 4 and 5, there were no differences in mean weekly dose of NeoRecormon between study groups. However, ferritin concentration increased from 280 ± 241 at baseline to 506 ± 405 at month 12 and no important differences in the groups were observed, while the other haematological parameters (haematocrit, haemoglobin) remained stable during the entire study. The frequency of blood transfusion and total volume of blood in group C were lower than in groups A and B and probably contributed to the rise in serum ferritin together with intravenous iron supplementation. There was no evidence of excessive iron load, as we are fully aware of the fact that iron overdose might also contribute to adverse outcomes in randomized trials of anaemia correction in CKD as stressed by van Buren et al. [13]. During prospective 12-month follow-up, 23 (5.8 %) of the patients died and 35 (8.8 %) were transplanted. No differences in death or transplantation rate were observed among groups A, B and C. The number of patients with adverse events, serious adverse events or drug-related adverse events in all groups was similar. As shown previously, at the start of dialysis, mean haemoglobin value is lower than at the diagnosis of the disease (respectively, 9.26 ± 1.7 vs 10.3 ± 2.3 g/dL) [12]. At the beginning of ESA therapy, mean haemoglobin value is 9.24 ± 1.4 g/dL close to the value in the TREAT study in diabetic CKD population [10]. During all subsequent study visits, mean haemoglobin value in the whole study group was stable and varied from 11.04 to 11.26 g/dL, again perfectly fitted in the new postulated target. Group A patients entered the study with lower haemoglobin concentration than the remaining groups; however, the differences shortly disappeared. During the first 3 months of the study, B patients had higher haematocrit than the remaining groups. Our study was designed before the results of the TREAT trial were available. Presented data reflect reimbursement policy in EEC. No differences in adverse events profile including serious adverse events, drug-related adverse events or cardiovascular events were observed among groups A, B and C. The death rate and kidney transplantation rate in all groups were similar. Therefore, our data support the concept that the adequate erythropoietin treatment does not enhance the risk of cardiovascular complications in HD population. Since our study was performed only in the EEC, therefore, it is very difficult to discuss our findings with other trials involving Europe (both Western and Eastern), USA or being global as TREAT. In the ORAMA trial prevalent, HD or CKD patients were randomized to either standard clinical care or computerized clinical decision support, and comparison between current management of renal anaemia in both CKD and dialysis patients in Eastern and Western European countries was performed [14]. In the GAIN study [15] involving only HD subjects treated with any ESA for at least 12 weeks, and for 18 months with epoetin beta, there were regional differences between Balkan, Baltics, and Eastern, Southern and Western Europe. However, these data were not analysed.

Reasonable dose of ESA to keep Hb target of 10 or 10.5 g/dL or a range of 10–12 g/dL has recently been postulated [16] although more radical position has been taken by Ajay K Singh, the principal investigator of the CHOIR study [9]. He suggests that avoiding use of ESAs in managing anaemia in non-dialysis patients with CKD is now the soundest approach [17]. An individualized approach to every patient and a proper control of increase in Hb, protecting from overshooting (not exceeding Hb 12 g/dL) may be a reasonable approach in most of patients with CKD. However, in our study, we found that 7 % (range from 0 to 11 %) occasionally had Hb >13 g/dL, which required caution. At the last two visits, none of the patient had Hb over 13 g/dL, whereas 17 % of the patients had Hb below 10 g/dL. In the prospective observational study RISCAVID study (‘RISchio CArdiovascolare nei pazienti afferenti all’Area Vasta In Dialisi’), performed on the 753 prevalent HD patients in the north-western area of Tuscany, Italy, the impact of haemoglobin levels, as continuous or categorical variable, on fatal and non-fatal CV events was not statistically significant [18]. However, in non-adjusted analysis, haemoglobin levels <11 g/dL were associated with the highest risk for all-cause mortality and fatal/non-fatal CV events, while patients with haemoglobin levels >11 g/dL had the lowest all-cause mortality risk which was comparable to the reference group (no ESAs). In the RISCAVID study focused on the ESA resistance and its relation to CV events, they found that ESA responsiveness can be considered a strong prognostic factor in HD patients and seems to be tightly related to protein-energy wasting and inflammation. In our study, we looked for the possible relations between early versus late ESA treatment and CV events. Our data suggest that predialysis ESA treatment have virtually no effect on the anaemia practice pattern in prevalent HD population in EEC. The early versus late ESA therapy in CKD does not influence the rate of CV events and mortality in prevalent HD population in EEC. The strength of our study is the fact that this is the largest and the most recent study performed in EEC, with similar reimbursement policy in these 3 countries. Furthermore, we performed a study on relatively homogeneous for race, geography, medical care and HD management population. Before TREAT results became available, we started to treat anaemia relatively late with low doses of ESA providing iron supplementation in vast majority of patients. Last January in USA the bundling system, which is similar to our reimbursement policy, was introduced. As reported at the Annual Dialysis Congress in San Antonio in February 2012, the use of ESA dropped significantly together with a rise in iron. In addition, percentage of Hb levels over 13 g/dL declined significantly, while Hb below 10 g/dL remained stable (personal communication). Our results indicate that relatively low use of ESA together with iron supplementation may yield expected results, that is, achieved Hb levels with the lowest possible adverse events, particularly cardiovascular. However, we are not aware of the pharmacoeconomic analysis till date. Moreover, our ERI was much lower than that reported for the US population by Kotanko et al. [19]. They also stated that lower ERI was associated with better anaemia control.

Our study has some important limitations. Of note is the very low mortality in our population studied, 5.7 % in the whole group and low number of all CV events after 12 months. In the RISCAVID study, the mortality after 36 months was 27.5 % and 208 out of 753 patients experienced a fatal/non-fatal CV event. Moreover, this was not a randomized trial, and potential confounders might have influenced our results. Previously, we reported a retrospective and baseline data, and we stressed the differences at the baseline among 3 groups studied as defined per protocol [12]. We would like to stress that vast majority of the patients studied had a-v fistula as their vascular access (*n* = 320), only 27 had permanent catheter and 38 had temporary catheter (for 6 patients no data are available). There were no grafts as vascular access in the population studied. It may also contribute to the low mortality, as well as the fact that patients enroled in clinical trials are more compliant and healthier than the prevalent patients in every HD unit.

In conclusion, ESA therapy increased haemoglobin concentration and no major differences in haematological parameters among the groups were observed during the entire study irrespective of early versus late start. Duration of ESA treatment before HD does not affect mortality, cardiovascular events or other adverse events among the groups during the observation period. Adequate anaemia treatment is the most sound approach yielding the best desirable outcomes.

## Appendix

*Latvia*: Regina Baufale, Natalija Bidzina,Inara Busmane, Vera Grotkere, Tatjana Kozlova, Guna Legzdina, Linda Micule, Gita Saumane-Baza, Aldis Spudass, Ligita Zepa.

*Poland*: Danuta Antczak-Jędrzejczak, Hanna Augustyniak-Bartosik, Wacław Bentkowski, Alicja Brylowska-Markowicz, Edward Ciechanowicz-Lewkowicz, Krzysztof Dziewanowski, Jacek Felisiak, Piotr Firczyk, Mirosław Grzeszczyk, Zbigniew Hruby, Marzena Janas, Krzysztof Jarzębski, Marian Klinger, Arkadiusz Lewartowski, Justyna Matulewicz-Gilewicz, Olech Mazur, Danuta Ostrowska-Reguła, Marita Piechowska, Jan Pulchny, Roman Radziszewski, Bolesław Rutkowski, Jacek Sobolewski, Roman Stankiewicz, Anna Struś, Arkadiusz Szwedowicz, Ewa Trafidło, Jerzy Wiatrow, Rafał Wnuk, Danuta Zaremba-Drobnik, Danuta Zwolińska.

*Serbia*: Jovan Bakovic,Ivana Budosan,Vidojko Djordjevic, Rosa Jelacic, Dragisa Jevremovic, Ljiljana Komadina, Djoko Maksic, Igor Mitic, Vidosava Nesic, Svetlana Pejanovic, Steva Pljesa, Nenad Rakic, Miomir Stojanovic, Ljubisa Veljancic.
